# Identification of Modulators of the Nuclear Receptor Peroxisome Proliferator-Activated Receptor α (PPARα) in a Mouse Liver Gene Expression Compendium

**DOI:** 10.1371/journal.pone.0112655

**Published:** 2015-02-17

**Authors:** Keiyu Oshida, Naresh Vasani, Russell S. Thomas, Dawn Applegate, Mitch Rosen, Barbara Abbott, Christopher Lau, Grace Guo, Lauren M. Aleksunes, Curtis Klaassen, J. Christopher Corton

**Affiliations:** 1 National Health and Environmental Effects Research Lab, US-EPA, Research Triangle Park, North Carolina, United States of America; 2 Hamner Institutes for Health Sciences, Research Triangle Park, North Carolina, United States of America; 3 RegeneMed, San Diego, California, United States of America; 4 Rutgers University, Ernest Mario School of Pharmacy, Department of Pharmacology and Toxicology, Piscataway, New Jersey, United States of America; 5 University of Washington, Seattle, Washington, United States of America; INRA, FRANCE

## Abstract

The nuclear receptor family member peroxisome proliferator-activated receptor α (PPARα) is activated by therapeutic hypolipidemic drugs and environmentally-relevant chemicals to regulate genes involved in lipid transport and catabolism. Chronic activation of PPARα in rodents increases liver cancer incidence, whereas suppression of PPARα activity leads to hepatocellular steatosis. Analytical approaches were developed to identify biosets (i.e., gene expression differences between two conditions) in a genomic database in which PPARα activity was altered. A gene expression signature of 131 PPARα-dependent genes was built using microarray profiles from the livers of wild-type and PPARα-null mice after exposure to three structurally diverse PPARα activators (WY-14,643, fenofibrate and perfluorohexane sulfonate). A fold-change rank-based test (Running Fisher’s test (p-value ≤ 10^-4^)) was used to evaluate the similarity between the PPARα signature and a test set of 48 and 31 biosets positive or negative, respectively for PPARα activation; the test resulted in a balanced accuracy of 98%. The signature was then used to identify factors that activate or suppress PPARα in an annotated mouse liver/primary hepatocyte gene expression compendium of ~1850 biosets. In addition to the expected activation of PPARα by fibrate drugs, di(2-ethylhexyl) phthalate, and perfluorinated compounds, PPARα was activated by benzofuran, galactosamine, and TCDD and suppressed by hepatotoxins acetaminophen, lipopolysaccharide, silicon dioxide nanoparticles, and trovafloxacin. Additional factors that activate (fasting, caloric restriction) or suppress (infections) PPARα were also identified. This study 1) developed methods useful for future screening of environmental chemicals, 2) identified chemicals that activate or suppress PPARα, and 3) identified factors including diets and infections that modulate PPARα activity and would be hypothesized to affect chemical-induced PPARα activity.

## Introduction

Coordinated efforts are underway by a number of agencies that regulate chemicals to define networks of adverse outcome pathways (AOP) [1, 2]. AOPs are defined as a series of mechanistically-linked key events starting with a molecular initiating event (MIE) in which a chemical interacts with a target culminating in an adverse outcome in a tissue. A group of AOPs that converge on liver cancer involve the activation of transcription factors that affect hepatocyte growth. The MIE of one of these AOPs is the activation of the nuclear receptor peroxisome proliferator-activated receptor α (PPARα). PPARα is activated by peroxisome proliferator chemicals (PPCs), a large class of structurally heterogeneous pharmaceutical and industrial chemicals originally identified as inducers of the size and number of peroxisomes in rodent livers. The PPAR family includes three family members (PPARα, β, and γ). The PPARα subtype plays a dominant role in mediating the effects of hypolipidemic and xenobiotic PPCs in the liver [3]. PPARα activation results in a predictable set of phenotypic responses in the livers of rats and mice, including the short-term responses of hepatocyte peroxisome proliferation, hepatomegaly, and hepatocyte hyperplasia. PPARα regulates a large battery of peroxisome assembly and fatty acid oxidation genes including those involved in the therapeutic hypolipidemic effects of PPARα targeted drugs. Under chronic exposure conditions, rats and mice exhibit an increased incidence of liver tumors [4]. These responses require a functional PPARα, because PPARα-null mice exposed to the PPARα agonists WY-14,643 (WY) or bezafibrate lack all of these short- and long-term responses [5–7]. Based on a large body of work, the mechanism by which liver tumors are induced by PPARα activators in rats and mice is generally thought to be irrelevant to humans [4].

Suppression of the ability of PPARα to activate fatty acid catabolism genes can lead to the buildup of fat in hepatocytes. Fatty liver disease is the most common liver disease in humans and encompasses a spectrum of hepatic steatosis which can progress to an inflammatory state (steatohepatitis) sometimes leading to cirrhosis and hepatocellular carcinoma [8]. Fatty liver disease occurs in people with excess alcohol consumption (alcoholic fatty liver disease) and people who are obese with and without added insulin resistance (non-alcoholic fatty liver disease) [9]. Fatty liver disease is often the result of the complex combination of increased energy uptake, increased hepatic lipogenesis, decreased energy combustion and decreased hepatic secretion of liver triglycerides. PPARα-null mice have provided valuable clues regarding the role of PPARα in energy balance in the liver and susceptibility to steatosis. PPARα-null mice are highly susceptible to fasting-induced steatosis and hyperlipidemia [10–12]. These mice develop severe steatohepatitis compared to wild-type mice when maintained on a diet deficient in methionine and choline [13], or when administered ethanol [14], implying a role for decreased fatty acid oxidation in the progression of steatosis to steatohepatitis. Aged PPARα-null mice on standard diets exhibit chronic steatosis, steatohepatitis and increases in combined hepatocellular adenomas and carcinomas [15, 16]. Thus, an AOP leading to steatosis and steatohepatitis involves suppression of PPARα activity and accumulation of fats due to decreases in fatty acid catabolism.

The ability to accurately predict PPARα activation or suppression would be useful to evaluate the potential for diverse factors including chemicals to contribute to liver cancer or steatosis by affecting PPARα. In the present study, a screen of a gene expression compendium was carried out to identify chemicals and other factors that affect PPARα activation. To facilitate screening, a gene expression signature used to predict PPARα activation or suppression was developed by identifying genes that were consistently altered by three PPARα activators in wild-type but not PPARα-null mice. Coupled with a fold-change rank-based similarity test, the signature was found to be very accurate in predicting activation of PPARα and was used to screen a mouse liver compendium of ~1850 comparisons to find chemicals and other factors that affect PPARα. Our screen identified chemicals, diets and infections that activate or suppress PPARα and could potentially contribute to liver cancer or steatosis, respectively.

## Methods

### Strategy for identification of factors that affect PPARα

**Fig 1:**
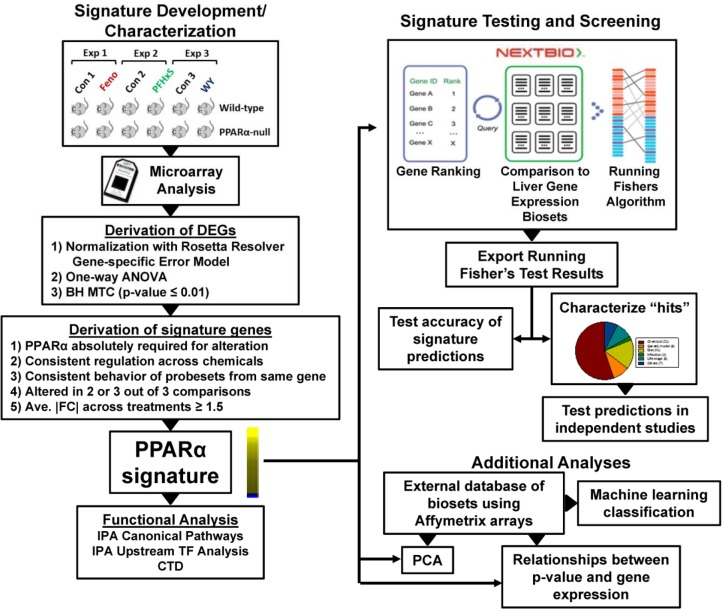
PPARα signature development/characterization and screening of a mouse liver gene expression compendium. Left, signature development and characterization. Wild-type and PPARα-null mice were treated with fenofibrate (Feno) [21], perfluorohexanesulfonate (PFHxS) (GSE55756), or WY-14,643 (WY) [22] in separate experiments carried out in three different labs. Differentially expressed genes (DEGs) were identified using Rosetta Resolver as indicated. Signature genes were identified from the DEGs after applying a number of filtering steps. Genes in the signature were evaluated by Ingenuity Pathway Analysis (IPA) for canonical pathway enrichment and potential transcription factor regulators and by the Comparative Toxicogenomics Database (CTD) to evaluate literature evidence for consistent regulation of signature genes by PPARα activators. Right, signature testing and screening. The PPARα signature was imported into the NextBio environment in which internal protocols rank ordered the genes based on their fold-change. Screening was carried out by comparison of the signature to each bioset using a pair-wise rank-based enrichment analysis (the Running Fishers algorithm). The results of the test including the direction of correlation and p-value for each bioset in the compendium were exported and used to populate a master table containing bioset experimental details. A test of the accuracy of the signature predictions was carried out with treatments that are known positives and negatives for PPARα activation. Screening “hits” were characterized, and a number of predictions were tested in independent studies. Additionally, an external gene expression database of experiments using Affymetrix gene chips was used in a machine learning classification analysis by BRB Array Tools, principal components analysis (PCA), and examination of the relationships between p-value and gene behavior. Part of the figure was adapted from a figure in [27].

The methods used in this study are outlined in **Fig. 1**. A screen for PPARα modulators required 1) a gene expression signature of PPARα-dependent genes, and 2) an annotated database of gene expression profiles (also called biosets) of statistically filtered genes. The PPARα signature is a list of genes with associated fold-change values that reflect average differences in expression between control and chemically-treated mice and that require PPARα for these changes. A commercially available gene expression database provided by NextBio (www.nextbio.com) facilitiated screening. The NextBio database contains over 110,000 lists of statistically filtered genes from over 15,700 studies carried out in 16 species (as of June, 2014). Each list (bioset) is compared to all other biosets in the database using a fold-change rank-based statistical test called the Running Fishers test which allows an assessment of the overlap in regulated genes and whether those genes are regulated in a similar or opposite manner. In this study, ~1850 biosets from mouse liver, mouse primary hepatocytes or hepatocyte-derived cell lines were evaluated. Available information about each bioset was extracted from NextBio and used to populate a database of information about the experiments. Each bioset was further annotated using information derived from the GEO submission and/or the original publication. Importantly, each bioset was annotated for the type of factor (e.g., chemical) and the name of the factor (e.g., WY-14,643) examined. To assess PPARα activation or suppression, the PPARα signature was uploaded to the Nextbio database and compared to all biosets in the database using the Running Fishers test. Results of the test were exported and used to populate the annotated compendium with p-values of each comparison. Test results were used to determine the accuracy of predictions and to further examine those biosets that achieved statistical significance. A number of predictions were tested in independent studies. Additional analyses were carried out using an independently-derived database of biosets used for PCA, determination of the relationship between Running Fisher’s p-value and behavior of genes in the signature, and machine learning classification.

### Animal studies

Three animal studies carried out as part of this investigation.

Study 1. Perfluorononanoic acid (PFNA) and perfluorohexane sulfonate (PFHxS) in wild-type and PPARα-null mice.

The animals study was carried out at the Reproductive Toxicology Facility of the EPA in Research Triangle Park, NC. Studies were approved by the U.S. EPA ORD/NHEERL Institutional Animal Care and Use Committee. The facilities and procedures used followed the recommendations of the 1996 NRC “Guide for the Care and Use of Laboratory Animals”, the Animal Welfare Act, and the Public Health Service Policy on the Humane Care and Use of Laboratory Animals. Animals were housed in fully-accredited American Association for Accreditation of Laboratory Animal Care (AAALAC) facilities. PPARα-null mice (129S4/SvJae-*PPARα* ^tm1Gonz^/J, stock #003580) and wild-type mice (129S1/SvlmJ, stock #002448) were purchased from The Jackson Laboratory (Bar Harbor, ME) and maintained as an inbred colony on the 129/Sv background at the U.S. EPA, Research Triangle Park, NC. Randomized animals were housed 4 per cage and allowed to acclimate for a period of one week prior to the conduct of the study. Food (Prolab 5P00, RMH 3000) and filtered distilled water were provided *ad libitum*. Animal facilities were controlled for temperature (20–24°C), relative humidity (40–60%), and kept under a 12 hr light-dark cycle. PPARα-null and wild-type male mice at 6–9 months of age were dosed by gavage for 7 consecutive days with either 0, 3, or 10 mg/kg PFHxS, or 1 or 3 mg/kg PFNA (Sigma-Aldrich, St, Louis, MO) in water. PFHxS was kindly provided by 3M Corp (St. Paul, MN). Four biological replicates consisting of individual animals were included in each dose group used for microarray analysis. Dose levels reflected exposures that produce hepatomegaly in adult mice without inducing overt toxicity. All dosing solutions were freshly prepared each day. At the end of the dosing period, animals were euthanized by CO_2_ asphyxiation and tissue was collected from the left lobe of the liver for preparation of total RNA. Livers were removed 24-hrs after the last dose and were rapidly snap-frozen in liquid nitrogen and stored at -70°C until analysis.

Study 2. Pregnenolone-16alpha-carbonitrile (PCN) in wild-type and PXR-null mice.

The animal study was carried out at the University of Kansas Medical Center (Kansas City, KS) and was approved by KUMC Institutional Animal Care and Use Committees. Animals were housed in fully-accredited American Association for Accreditation of Laboratory Animal Care (AAALAC) facilities. Eight-week-old adult C57BL/6 mice were purchased from Jackson Laboratories (Bar Harbor, ME). PXR-null breeder pairs were engineered and backcrossed into the C57BL/6 background [17]. Adult male wild-type mice and PXR-null mice were maintained on Teklad Rodent Diet 8604 (Harlan, Madison, WI) and tap water *ad libitum*. Mice were housed 2 to 4 per cage. All mice were treated once a day i.p. with either vehicle (corn oil) or PCN at 400 mg/kg/day for 4 days. Livers were removed 24-hrs after the last dose. Portions of the livers were rapidly snap-frozen in liquid nitrogen and stored at -70°C until analysis.

Study 3. 12 treatment study in male and female mice.

This animal study was carried out at the Hamner Institute for Health Sciences, Research Triangle Park, NC. Animal use in this study was approved by the Institutional Animal Use and Care Committee of The Hamner Institutes for Health Sciences and was conducted in accordance with the National Institutes of Health guidelines for the care and use of laboratory animals. Animals were housed in fully-accredited American Association for Accreditation of Laboratory Animal Care (AAALAC) facilities. Aroclor 1260 was purchased from Ultra Scientific (Kingstown, RI). Ciprofibrate, β-naphthoflavone, cobalt chloride, lipopolysaccharide, phenobarbital, phenylhydrazine, and WY-14,643 (WY) were purchased from Sigma Aldrich (St. Louis, MO). Interleukin-6 (IL-6) and tumor necrosis factor-α (TNFα) were purchased from R&D Systems (Minneapolis, MN). 2,3,7,8-Tetrachlorodibenzo-*p*-dioxin (TCDD) and PCB-153 were purchased from NeoSyn (New Milford, CT). Six-week-old male and female B6C3F1 mice (B6C3F1/Crl) were purchased from Charles River Laboratories (Raleigh, NC). Upon arrival, the mice were acclimated for one week prior to initiation of the study. The animals were weighed and randomized into treatment groups to ensure the mean body weight in each group was approximately the same. Animals were housed three per cage except male mice which were housed individually in shoe box cages and segregated by treatment group. Alpha-dri cellulose bedding (Shepard Specialty Papers, Kalamazoo, MI) was used. Animals had access to reverse osmosis water (Hydro Systems, Durham, NC) and pellet NIH-07 certified feed (Zeigler Brothers, Gardners, PA) *ad libitum*. The animal room was kept within the standard temperature and humidity parameters (64 to 79°F with 30 to 70% relative humidity) and standard light cycle (0700–1900 hrs). The animal exposures were similar to those employed in a previous study [18]. Exposures of mice of both sexes were initiated at 7 weeks of age and were performed by the route, dose, and time listed in **S1 File**. A matched vehicle control was run concurrently with each exposure. Animal necropsy occurred at the time points specified in **S1 File**. Mice were weighed, anesthetized with Ketamine (90 mg/kg) and Xylazine (10 mg/kg), and exsanguinated to obtain blood. The liver was removed, weighed, and the left lobe fixed in 10% neutral buffered formalin. The remaining right, caudate, and median lobes were placed in RNA*later* (Ambion, Austin, TX).

### RNA isolation

Total RNA was isolated and purified from mouse livers according to the mirVana miRNA isolation kit (Ambion, Austin, TX), or Qiagen RNeasy or AllPrep Mini/Midi kits. The integrity of each RNA sample was determined using an Agilent 2100 Bioanalyzer (Agilent, Foster City, CA), and RNA quantity was determined using a Nanodrop ND-1000 (Thermo Fisher Scientific, Wilmington, DE).

### Microarray analyses

Microarray analyses were performed on the livers of mice as part of this study, and the raw data files and experiment descriptions are archived in GEO. These studies include PFNA and PFHxS in wild-type and PPARα-null mice, PCN in wild-type and PXR-null mice, and the 12 treatment study. Liver gene expression analysis was performed according to the Affymetrix recommended protocol using Affymetrix Mouse Genome 430 2.0 or 430A GeneChips. Total RNA (5 μg per sample) was labeled using the Affymetrix One-Cycle cDNA Synthesis protocol and hybridized to arrays as described by the manufacturer (Affymetrix, Santa Clara, CA). Microarray hybridizations were conducted overnight at 45°C while rotating in an Affymetrix hybridization oven. After 16 hrs of hybridization, the cocktail was removed and the arrays were washed and stained in an Affymetrix GeneChip fluidics station 450 according to the Affymetrix-recommended protocol. Arrays were scanned on an Affymetrix GeneChip scanner. The RNA from three or four mice per group were examined. Raw microarray files have been submitted to GEO (PFNA and PFHxS in wild-type and PPARα-null mice (GSE55756), PCN in wild-type and PXR-null mice (GSE55746), and the 12 treatment study (GSE55084)).

### Identification of differentially expressed genes in microarray datasets

All of the Affymetrix .cel files from the above studies were first evaluated by Bioconductor SimpleAffy [19] to assess sample quality. Cel files from individual studies were normalized using Rosetta Resolver version 7.1 Affymetrix Rosetta-Intensity Profile Builder software (Rosetta Inpharmatics, Kirkland, WA). The cel files in each study were normalized as a group. Statistically significant genes were identified by one-way ANOVA with a false discovery rate (Benjamini-Hochberg test) of ≤ 0.01. In addition to the studies described here, a number of publicly available studies were analyzed using the same methods to derive statistically significant gene lists or biosets (i.e., gene expression differences between two conditions) (to give a total of ~890 biosets). Publically available sources included Gene Expression Omnibus (GEO) and ArrayExpress. Tables were built using the common probe sets shared by 430A or 430_2 chip types. Only studies that evaluated gene expression in mouse liver, mouse primary hepatocytes, or hepatocyte-derived cell lines was used to build the ~890 bioset database.

### Classification analysis

Analyses were performed using BRB-ArrayTools version 4.2.1 Stable Release developed by Dr. Richard Simon and BRB-ArrayTools Development Team (http://linus.nci.nih.gov/BRB-ArrayTools.html) [20]. All samples used in the training and testing sets were first log2 normalized using RMA in the RMAExpress software environment (http://rmaexpress.bmbolstad.com/). The cel files from the three Affymetrix array types (mouse 430A, mouse 430_2 and mouse 430PM arrays) were normalized separately. Normalized expression values of common probesets (22,626) were then combined into one master file. Prior to classification, probesets were excluded under any of the following conditions: 1) minimum fold change—less than 20% of the expression data values have at least a 1.5-fold change in either direction from the genes median value, 2) variance is in the bottom 75^th^ percentile, or 3) percent missing exceeds 50%. Filtering using these criteria resulted in 5644 probesets used in the classification study. The 7 models used for class prediction included Compound Covariate Predictor, Bayesian Compound Covariate, Diagonal Linear Discriminant Analysis, 1- and 3-Nearest Neighbor Classifications, Nearest Centroid, and Support Vector Machines with Linear Kernel. The models incorporated genes that were differentially expressed at p ≤ 0.001 significance level as assessed by the random variance t-test. The prediction error of each model was estimated using 10-fold cross-validation. Two training sets were used for predicting PPARα activation. The first training set consisted of samples from wild-type and PPARα-null mice and included 64 positive samples and 115 negative samples. The second training set consisted of the same set but lacked the control and treated PPARα-null samples and included 64 positive samples and 20 negative samples. The derived classifiers of 179 and 534 probesets for training sets including or excluding the PPARα-null samples, respectively were then used to predict PPARα activation of the remaining samples. A test set of samples known to be positive or negative, respectively for PPARα activation came from a number of studies in which mice or mouse primary hepatocytes were exposed to structurally diverse PPARα activators or control substances.

### Construction of a PPARα-dependent gene signature

Methods for signature development are outlined in **Fig. 1, left**. A list of probe sets that comprise the PPARα signature was derived from the comparisons using livers of wild-type and PPARα-null mice treated with fenofibrate for 6 hrs [21], WY for 5 days [22] or PFHxS (10 mg/kg/day) for 7 days (GSE55756; Rosen et al., in preparation). The strategy was similar to that reported to find genes regulated by SREBP1 [23] although with stricter criteria. The strategy is also analogous to that used by Bild et al. (2006) [24] to find genes regulated by oncogenic factors in cancer cell lines. Three statistical tests were used to generate each of the three lists of PPARα-dependent genes. Gene lists were derived from chemical treated wild-type mice compared to wild-type controls and chemical treated PPARα-null mice compared to corresponding PPARα-null controls. Genes were identified that exhibited differences in expression in wild-type mice but no statistically significant expression in the same direction in the PPARα-null mice. This list of genes was then examined for statistical differences between the treated wild-type mice and the treated PPARα-null mice. The three lists of genes (one for each chemical) were compared and probe sets were selected based on the following criteria: 1) probe sets exhibited the same direction of change, 2) probe sets that encoded the same gene had identical direction of change after exposure, 3) probe sets were altered in 2 or 3 out of the 3 comparisons, 4) the │average fold-change for each probe set│ was ≥ 1.5-fold, and 5) the probe sets were not also altered in the same direction in gene expression signatures for AhR, CAR, Nrf2, and STAT5b (Oshida et al., in preparation). These criteria for selection of the probe sets insured that the probe sets met the following characteristics: absolute dependence on PPARα, robust response, and consistent chemical-independent regulation. The final list of probesets and gene information in the PPARα signature is found in **S2 File**.

### Functional analysis of PPARα signature genes

The Comparative Toxicogenomics Database (CTD; http://ctdbase.org/) was used to find annotated relationships between chemicals and genes. Only the annotations “decreases^expression”, or “increases^expression” were used. A table was built comprised of genes in the PPARα signature and annotations from CTD using common gene abbreviations. The full list of genes in the PPARα signature was analyzed using the IPA canonical pathway and upstream analysis functions. All results were exported as excel files and filtered based on p-value.

### Annotation of a mouse liver gene expression compendium

All of the biosets examining gene expression in mouse liver, mouse primary hepatocytes or mouse liver-derived cell lines were annotated for study characteristics allowing systematic assessment of the effect of different factors on PPARα activity. The list of descriptors provided for each of the biosets included study ID (i.e., source), contrast name, class of perturbation (e.g., chemical, diet, genotype, etc.), chemical or treatment, CAS#, sex, source, array type, and type of genetic model used (e.g., wild-type, knockout/knockdown or transgenic). The entire list of biosets which exhibit PPARα activation or suppression discussed in this study is found in **S2 File**. Detailed descriptions of each experiment are available at GEO (http://www.ncbi.nlm.nih.gov/geo/) or ArrayExpress (http://www.ebi.ac.uk/arrayexpress/) if archived there or in the original publications. All biosets in the compendium were compared to the signature. Resultant p-values were converted to –log10 values. Those comparisons which exhibited negative correlation to the signature were converted to a negative value. Results of the Running Fisher’s test (i.e., comparisons of the PPARα signature to each bioset in NextBio) were exported and used to populate the database with p-value and correlation direction for each bioset.

### Classification prediction of PPARα activation using the Running Fisher’s Test

A rank-based nonparametric analysis strategy called the Running Fisher’s algorithm within the NextBio database environment (http://www.nextbio.com/) was used for evaluating PPARα activation. The Running Fisher’s algorithm is analogous to the Gene Set Enrichment Analysis (GSEA) method [25, 26]. The Running Fisher’s algorithm differs from GSEA in the assessment of the statistical significance, as p-values are computed by a Fisher's exact test rather than by permutations (see Kuperschmidt et al., 2010 for details [27]). This normalized ranking approach enables comparability across data from different studies, platforms, and analysis methods by removing dependence on absolute values of fold-change, and minimizing some of the effects of normalization methods used, while accounting for the level of genome coverage by the different platforms. The PPARα signature was imported into NextBio and used without any further filtering. Biosets from microarray experiments in which the PPARα activation state was known were manually curated from studies that examined the transcriptional effects of PPARα activators (Currie et al., 2005 [28]; Schaap et al., 2012 [29]; Study 1 and Study 3 (above)). The final number of biosets evaluated was 39 positives and 24 negatives. Unlike the 7 machine learning classification methods described above, conditions for classification were not derived from gene behavior as the signature was fixed. A p-value ≤ 10^-4^ was selected as our cutoff for significance. In preliminary studies, signatures for three transcription factors (i.e., AhR, CAR, PPARα) were constructed using similar methods. The p-values from the Running Fisher’s test for each of the three signatures across the ~1850 biosets were subjected to a Benjamini-Hochberg multiple test correction of α = 0.001 resulting in a corrected p-value = 9.4E-5 that was rounded to a p-value = 1E^-4^. Classification tests were subsequently performed using this cutoff.

### Additional computational analyses

Principal components analysis (PCA) was evaluated using unfiltered fold-change values of the PPARα signature probe sets from biosets examined by Affymetrix 430_2 or 430A chips using Eisen Lab Cluster (http://rana.lbl.gov/EisenSoftware.htm) and visualized by SigmaPlot. Heat maps were generated using Eisen Lab Cluster and Treeview software (http://rana.lbl.gov/EisenSoftware.htm).

### Evaluation of Selected Genes by Real-Time RT-PCR

Livers used for the RT-PCR experiments were from the following studies: WY (50 mg/kg/day for 3 days) and AGN (3 mg/kg/day for 3 days) in wild-type and PPARα-null mice [30], phenobarbital (0.085% w/w diet for 28 days) in wild-type and CAR-null mice [31], TCDD (one 100 μg/kg injection and sacrifice 2 weeks later) in wild-type and AhR-null mice [32], oltipraz (75 mg/kg/day for 4 days) in wild-type and Nrf2-null mice [33], and GW4064 (150 mg/kg 3X a day for one day) in wild-type and FXR-null mice [65]. Additional studies used for RT-PCR include Study 1 and 2 described above. The levels of expression of selected genes were quantified using real-time reverse transcription-PCR (RT-PCR) analysis. Briefly, total RNA was reverse transcribed with murine leukemia virus reverse transcriptase and oligo(dT) primers. The forward and reverse primers were designed using Primer Express software, version 2.0 (Applied Biosystems, Foster City, CA). The SYBR green DNA PCR kit (Applied Biosystems, Foster City, CA) was used for real-time PCR analysis. The relative differences in expression between groups were expressed using cycle threshold (Ct) values as follows. The Ct values of the genes were first normalized with β–actin and glyderaldehyde 3-phosphate dehydrogenase (GAPDH) of the same sample. Assuming that the Ct value is reflective of the initial starting copy and that there is 100% efficiency, a difference of one cycle is equivalent to a two-fold difference in starting copy. Means and S.D. (*n* = 3–5) for RT-PCR data were calculated by Student's or Aspin-Welch’s *t*-test. The level of significance was set at *p* ≤ 0.05.

## Results and Discussion

### Classification analysis using machine learning algorithms

PPARα activation was predicted using 7 classification models as detailed in the Methods section. To determine the contribution of PPARα-null samples on prediction, two training sets were used in the prediction models including the samples from livers of wild-type and PPARα-null mice treated with chemical and synthetic triglyceride activators ([22, 34, 35] Study 1 in Methods) and the same dataset lacking the control and treated PPARα-null samples. The derived classifiers of 179 and 534 probesets, respectively were then used to predict PPARα activation of test samples. An independent manually curated test set of 66 and 224 samples positive or negative, respectively for PPARα activation came from studies in which mice or mouse primary hepatocytes were exposed to PPARα activators or vehicle controls. The models using the wild-type and PPARα-null samples in the training set had excellent sensitivity (sensitivity range, 99–100%; mean, 99%) but had low specificity (specificity range, 67–76%; mean, 71%) (**S1 File**). The models using only the wild-type samples as the training set had similar sensitivity compared to using all samples (range, 92–100%; mean, 98%) and similar specificity (range, 61–77%; mean, 68%), indicating that the wild-type vs. null comparisons of chemical treatments do not improve accuracy in classification predictions. Because of the less than ideal specificity, none of the models were thought to be adequate for predicting PPARα activation of additional samples.

### Construction and characterization of the PPARα signature

Other strategies were considered to identify PPARα activation in genomic databases. A rank-based enrichment analysis strategy called the Running Fisher’s algorithm [27] was evaluated for prediction. The Running Fisher’s test is analogous to the Gene Set Enrichment Analysis (GSEA) method [25, 26]. As originally described by Lamb et al. (2006) [25], rank-based enrichment statistics allows a gene signature to be compared to large collections of high-throughput data to find associations with diseases and drug treatments. Fold-change, as a ranking metric, has been shown to have greater concordance than p-values from statistical tests as fold-change rank is platform-independent [36].

**Fig 2:**
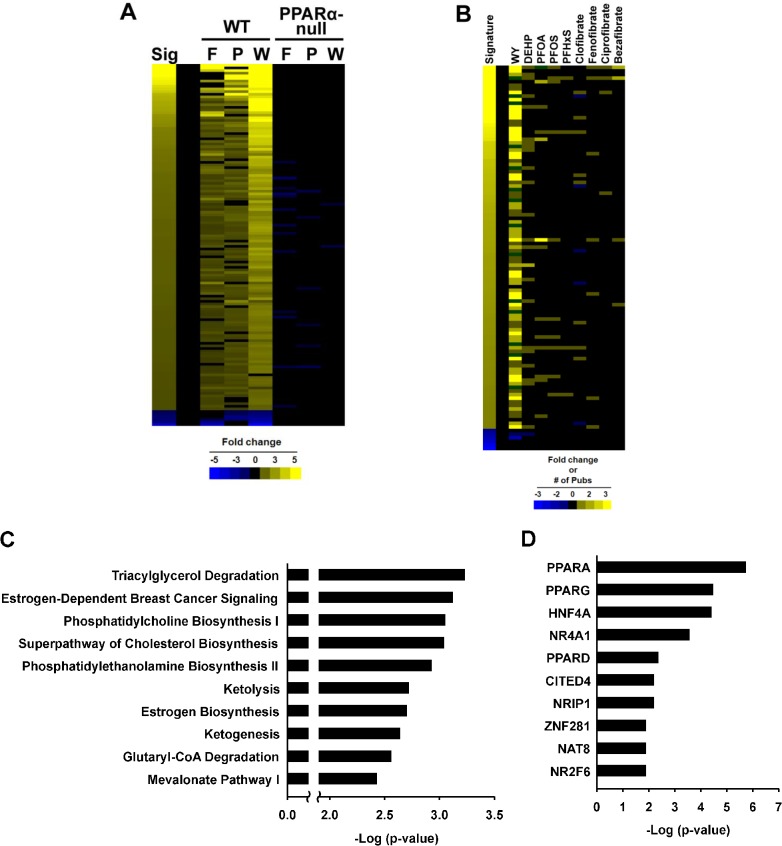
Characterization of the PPARα signature. A. Expression behavior of genes in the final signature. The heat map shows the expression of the 147 probe sets after exposure to fenofibrate (F), PFHxS (P), and WY-14,643 (W) in wild-type and PPARα-null mice compared to the signature (Sig). B. Expression behavior of the PPARα signature genes from the Comparative Toxicogenomics Database (CTD). Expression is shown as increased (yellow) or decreased (blue). If two or more references indicate expression in both directions, the gene is represented by green. The intensity of the signature was determined by fold-change, whereas the intensity of the genes in the 9 chemical comparisons reflects the number of publications that found the gene to be altered. C. Top canonical pathways significantly enriched by the genes in the PPARα signature. Genes were examined by Ingenuity Pathways Analysis. D. Transcription factors predicted to regulate the genes in the PPARα signature as determined by Ingenuity Pathways Analysis. The 10 most significant transcription factors are shown.

To test the usefulness of the Running Fisher’s test, PPARα signature genes were identified as described in the Methods and Fig. 1 using microarray profiles from the livers of wild-type and PPARα-null mice treated with fenofibrate for 6 hrs [21], WY for 5 days [22] or PFHxS (10 mg/kg/day) for 7 days (this study). A total of 147 probe sets (137 with increased expression and 10 with decreased expression, collapsing to a total of 131 genes) were identified which exhibited similar regulation by two or three out of the three compounds. The full list of genes is found in **S2 File**. The expression of genes in the PPARα signature was examined in treated wild-type and PPARα-null mice for the three chemicals. **Fig. 2A** shows the complete dependence of chemical-induced expression on PPARα of the genes in the signature. A small number of genes that were increased in wild-type mice exhibited decreased expression in the PPARα-null mice after chemical exposure, although the changes were relatively minor (< |2-fold|) and did not exhibit consistent effects across the chemicals. Many of the signature genes are known targets of PPARα including *Pdk4* [22], *Acot8* [37] and *Slc27a4* (also known as fatty acid transporter 4 [38]). Notably absent were genes commonly considered specific PPARα targets including genes involved in β- or ω-oxidation of fatty acids such as *Acox1* and *Cyp4a* family members, *Cyp4a10*, and *Cyp4a14*. Our comparison of gene expression in similarly treated wild-type and PPARα-null mice showed that these genes were induced in the livers of both wild-type and PPARα-null mice by a number of PPARα activators (data not shown; described for perfluorooctanoic acid (PFOA) in [39]). Additional transcription factors including PPARβ and PPARγ subtypes may mediate the regulation of these genes in the absence of PPARα as has been noted before in studies in which PPARα-null mice were treated with PPARβ and PPARγ activators [40].

To determine whether the genes in the PPARα signature were previously identified as regulated by PPARα activators, the Comparative Toxicogenomics Database (CTD; http://ctdbase.org/) was used to find published relationships between chemicals and the signature genes in mice. Gene-chemical interactions for 9 known PPARα activators are shown in **Fig. 2B** across the 107 genes of the signature annotated in CTD. Most of the gene-chemical interactions have been annotated for WY, a commonly used reference chemical. Far fewer interactions have been annotated for the other 8 chemicals. In general, the direction of expression changes caused by exposure to the chemicals was consistent with the direction of changes of the genes in the signature. There were a number of examples of two or more citations in which a gene exhibited increased and decreased regulation (green) or regulation opposite to that of the signature. These inconsistencies in expression changes with the signature may be attributed to effects observed in tissues other than the liver as CTD does not document the tissue origin of the change. However, the vast majority of the PPARα signature genes exhibited directional changes in chemical-induced expression consistent with findings in the literature.

The PPARα signature genes were evaluated for canonical pathway enrichment by Ingenuity Pathway Analysis (IPA) (**Fig. 2C**). The top 10 pathways enriched with the signature genes included those previously associated with PPARα regulation including lipid and ketone body homeostasis [10–13]. The upstream analysis function of IPA predicted that a number of transcription factors regulate the signature genes (**Fig. 2D**). PPARα was the top scoring transcription factor (p-value = 1.90E-6). Other transcription factors included PPARβ/δ, PPARγ, NR4a1 (Nurr77), and HNF4α.

### Evaluation of the predictive accuracy of the PPARα signature

**Fig 3:**
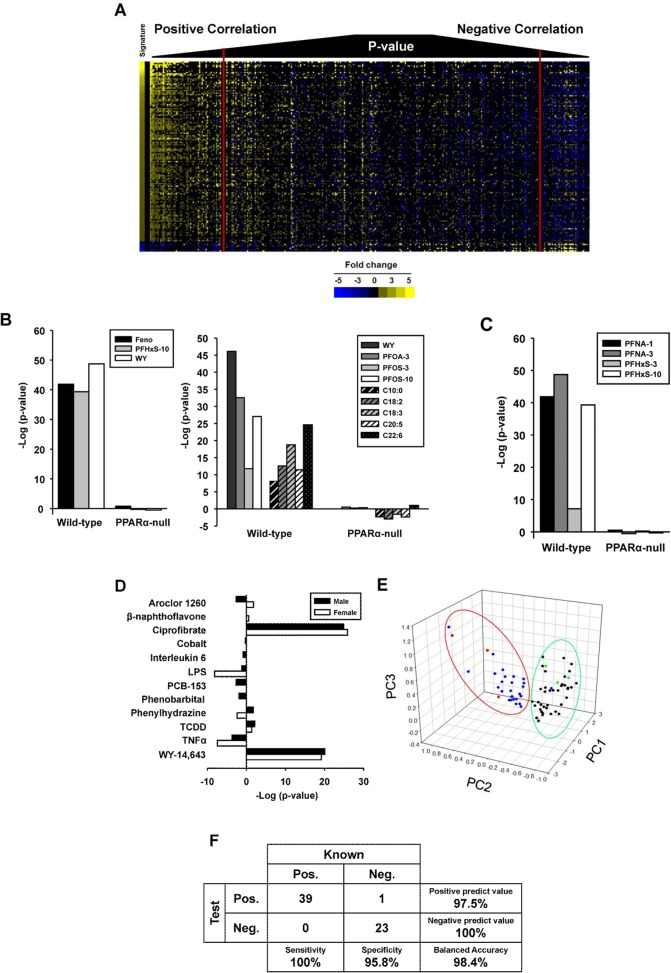
Prediction of PPARα activation using the Running Fisher’s algorithm. A. Heat map showing the expression of genes in the PPARα signature across 332 biosets. Biosets were ordered based on their similarity to the PPARα signature using the p-value from the Running Fisher’s algorithm. Biosets with positive correlation are on the left and biosets with negative correlation are on the right. The red vertical lines denote the position of biosets with a p-value = 10^-4^. B. (Left) Correlation of the PPARα signature to contrasts from the three chemicals used to derive the signature in wild-type but not PPARα-null mice. All p-values were converted to –log10 values. Those comparisons which exhibited negative correlation to the signature were converted to a negative value. (Right) Correlation of the PPARα signature to chemical and synthetic triglyceride activators of PPARα from wild-type but not PPARα-null mice. The compounds were WY-14,643 (WY) (GSE8396), PFOA at 3 mg/kg/day (PFOA-3) (GSE9786), PFOS at 3 or 10 mg/kg/day (PFOS-3, -10) (GSE22871) or synthetic triglycerides composed of the indicated fatty acids (GSE8396). C. The PPARα signature correctly identifies PFNA and PFHxS as PPARα activators in wild-type but not PPARα-null mice. Exposure to the indicated chemicals is described in Study 1 (Methods). D. The PPARα signature correctly identifies the two known PPARα activators (WY and ciprofibrate) in male and female mice exposed to 12 diverse treatments. See Study 3 in Methods for details of exposure conditions. E. The signature genes separate known positives and negatives for PPARα activation using principal components analysis. The first three principal components are shown, derived from the unfiltered expression changes of the signature genes. Red and green, the three chemical treatments in wild-type or PPARα-null mice used to derive the signature, respectively. Blue and black, chemicals or synthetic triglycerides in wild-type or PPARα-null mice, respectively. F. Summary of the sensitivity and specificity of the test for PPARα activation. The signature was compared to chemicals in wild-type or PPARα-null mice that were known positives or negatives for PPARα activation.

The signature was compared to gene lists using the Running Fisher’s algorithm resulting in a correlation direction (positive or negative) and an associated p-value of the similarity [27]. Because the Running Fisher’s test uses similarity as a metric, it could be hypothesized that biosets which are similar to each other (based on a low p-value) would “look” similar. To visualize the relationship between the Running Fisher’s test p-value and the expression of genes in the signature, 332 biosets of statistically filtered genes were evaluated for similarity to the PPARα signature and then sorted by p-value. The left of **Fig. 3A** shows that for biosets which had a positive correlation to the signature, the greater the statistical significance, the more the bioset “looks” like the signature due to similarities in the direction and the relative magnitude of the changes. These biosets included wild-type mice treated with a number of commonly recognized PPARα activators (di-2-ethylhexyl phthalate, fenofibrate, PFHxS, PFNA, PFOA, WY). The right of the figure shows a smaller group of biosets which exhibited negative correlation to the signature. The biosets on the far right are most significant for negative correlation and included a number of treatments (acetaminophen (APAP), lipopolysaccharide (LPS), tumor necrosis factor (TNF)α) as well as biosets in which PPARα-null mice were compared to the corresponding wild-type mice. In general, these biosets exhibited a pattern of expression that was opposite to that of the signature. Given that PPARα is known to be suppressed by a number of factors (see below), the biosets with negative correlation to the PPARα signature were hypothesized to reflect suppression of PPARα activation.

To examine the predictive behavior of the signature, biosets from wild-type and PPARα-null mice treated with chemicals and synthetic triglycerides were compared to the signature. As expected, the biosets from wild-type mice treated with the three compounds used to build the signature exhibited statistically significant similarity (p-values < 10^-38^), whereas the biosets from the corresponding treated PPARα-null mice were not significant (**Fig. 3B, left**). Likewise, examination of 4 additional chemical treatments and 5 synthetic triglyceride treatments not used in the construction of the signature showed the signature clearly distinguishes between treated wild-type and treated PPARα-null mice (**Fig. 3B, right**). For the synthetic triglycerides in wild-type mice, there was generally increasing significance with increasing fatty acid length, consistent with increased activation of PPARα in trans-activation assays with increasing chain length of the fatty acid [41].

Two separate animal studies were carried out to independently evaluate the ability of the signature to predict PPARα activation. In the first study, wild-type and PPARα-null mice were exposed to the known PPARα activators PFNA or PFHxS at two dose levels for 7 days. Treatments in all of the wild-type mice but none of the corresponding PPARα-null groups exhibited significant similarity with the signature (**Fig. 3C**). (The bioset of wild-type or PPARα-null mice treated with PFHxS at 10 mg/kg used to build the signature is also shown here for comparison.)

The second study was designed to determine whether the signature could distinguish between compounds that are known activators of PPARα from those that activate other transcription factors. Male and female wild-type mice were separately administered 12 different chemicals or biological agents. These included those that primarily activate AhR (TCDD, beta-naphthoflavone), CAR (arochlor-1260, PCB-153, phenobarbital), or PPARα (ciprofibrate, WY). Other treatments were expected to induce an inflammatory response (LPS, interleukin-6, tumor necrosis α (TNFα)) or hypoxia (cobalt, phenylhydrazine). Ciprofibrate and WY in males and females activated PPARα whereas the other treatments did not (**Fig. 3D**). LPS and TNFα caused suppression of PPARα in females but not males. An inflammatory state has been shown to suppress PPARα activity under some conditions ([41] and discussed below). Thus, the PPARα signature correctly identified compounds which activate or repress PPARα from those that do not.

Biosets of known positives and negatives including the ones discussed above were examined by principal components analysis (PCA) to determine whether the expression behavior of the genes in the PPARα signature can separate the two classes. **Fig. 3E** shows the clear separation between the known positives (blue and red dots within the red oval) and the known negatives (black and green dots within the green oval). Only one bioset in which PPARα was expected to be activated was negative (synthetic triglyceride C18:1 in wild-type mice) (GSE8396); however, this treatment resulted in only 20 significantly changed genes overall (data not shown) indicating there were minimal treatment-induced changes in gene expression.

A classification analysis was performed using the PPARα signature. Classification of activation required a p-value ≤ 10^-4^. The final number of biosets evaluated was 39 positives and 24 negatives. The signature predictions resulted in 100% sensitivity and 96% specificity, giving a balanced accuracy of 98% (**Fig. 3F**). The one false negative was from one unpublished study (GSE27948) in which mice were administered 10 mg/kg/day of WY. It should be noted that the positives used in the test set were all from animals or hepatocytes exposed to high concentrations of PPARα activators and would be expected to achieve close to maximal transcriptional responses. Future studies using a broad range of doses for weaker activators will be needed to rigorously determine the predictive accuracy of the methods in classifying chemicals at submaximal levels of activation. However, evaluation of the predictive power of the signature resulted in an excellent balanced accuracy.

### Behavior of PPARα-regulated genes *Pdk4* and *Cyp4a10* in the livers of mice exposed to activators of AhR, CAR, FXR, Nrf2, PPARα, PXR and RXR

**Fig 4:**
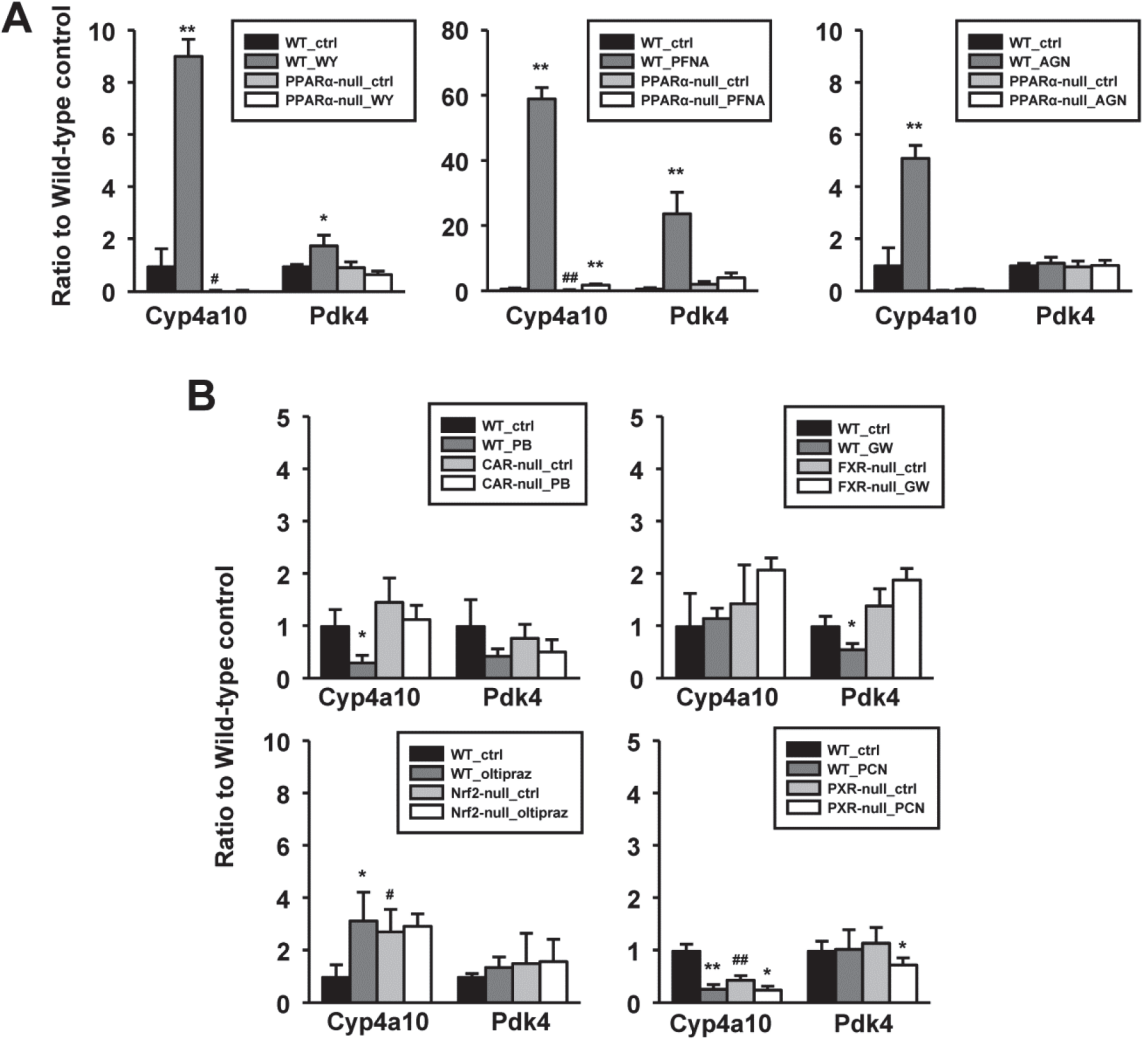
Expression of *Cyp4a10* and *Pdk4* genes in the livers of mice exposed to reference compound activators of CAR, FXR, Nrf2, PPARα, and PXR. Expression was examined in the livers from the indicated wild-type or nullizygous mice after exposure to a prototypical activator of that transcription factor. A. Expression behavior in wild-type and PPARα-null mice exposed to WY, PFNA or AGN194,204 (AGN). B. Expression behavior in wild-type and the indicated null mice after exposure to phenobarbital (PB), GW4064 (GW), oltipraz, or PCN. Significantly different from corresponding control: * p < 0.05, **p < 0.01. Significantly different between controls in wild-type and nullizygous mice: ^#^ p < 0.05, ^##^ p < 0.01.

The expression of two PPARα-regulated genes was examined across a number of treatments to determine the specificity of the expression pattern and for evaluation of their usefulness as biomarkers of PPARα activation. The expression of the PPARα signature gene *Pdk4* was compared to *Cyp4a10*, typically increased by PPARα activators and often used as a “specific” marker gene for PPARα activation. However, as described above in constructing the PPARα signature, *Cyp4a10* was found to be activated by PPARα activators in both wild-type and PPARα-null mice and thus was excluded from the PPARα signature. Expression was examined in the livers of wild-type mice and mice nullizygous for AhR, CAR, FXR, Nrf2, PPARα, and PXR treated with the prototypical activators TCDD, phenobarbital, GW4064, oltipraz, WY, and PCN, respectively. After exposure to WY, both genes exhibited increases in wild-type mice but no changes were observed in PPARα-null mice (**Fig. 4A**). Exposure to PFNA resulted in dramatic dose-dependent increases in both genes in wild-type mice. Minor but statistically significant increases in *Cyp4a10* but not *Pdk4* were also observed in PPARα-null mice, indicating that *Pdk4* but not *Cyp4a10* is a specific target for PPARα. Additionally, gene expression was examined in wild-type and PPARα-null mice after exposure to a panRXR agonist, AGN194,204 (AGN) which activates nuclear receptor-RXR heterodimers through RXR activation. Treatment with AGN resulted in activation of *Cyp4a10* in wild-type but not PPARα-null mice. In contrast, AGN treatment did not have an effect on *Pdk4* expression. These observations highlight differences in activation of the two genes; *Cyp4a10* was activated in the absence of PPARα and by RXR, whereas *Pdk4* was PPARα-dependent and not activated by RXR.

In contrast to the large inductions of *Cyp4a10* and *Pdk4* by PPARα activators, the other reference compounds had relatively minor effects on the expression of the two genes (**Fig. 4B)**. Of interest were the 1) suppression of *Cyp4a10* but not *Pdk4* by phenobarbital in wild-type but not CAR-null mice, 2) suppression of *Pdk4* but not *Cyp4a10* in wild-type mice with no change in hepatocyte-specific FXR-null mice by GW4064, 3) increases in *Cyp4a10* in wild-type mice by oltipraz and Nrf2-null control mice, but no change with oltipraz treatment in Nrf2-null mice, and 4) suppression of *Cyp4a10* in wild-type and PXR-null mice and *Pdk4* in PXR-null mice only by PCN. Robust and receptor-dependent inductions of marker genes occurred for CAR (*Cyp2b10*), Nrf2 (*Nqo1*), and PXR (*Cyp3a11*) as well as modest induction of a marker gene for FXR (*Prtn3*) (data not shown) indicating that absence of altered regulation of *Cyp4a10* and *Pdk4* was not due to absence of chemical-induced responses. Examination of the PPARα signature predictions showed that CAR and PXR inducers do not suppress PPARα, and the FXR activator does not modulate PPARα activation (data not shown). Thus, minor alterations in *Pdk4* expression by itself do not necessarily determine signature behavior.

### Screening for PPARα modulation in a mouse liver gene expression compendium

The PPARα signature and the Running Fisher’s algorithm were used for classifying biosets for effects on PPARα. The compendium of biosets examined consisted of gene expression changes in the livers of mice or primary hepatocytes by diverse factors. The compendium contains ~1850 biosets of gene expression changes between control and experimental states including ~470 chemical, ~450 gene, ~220 diet, ~100 hormone or cytokine, ~90 life stage, ~90 stress and ~120 strain comparisons.

**Fig 5:**
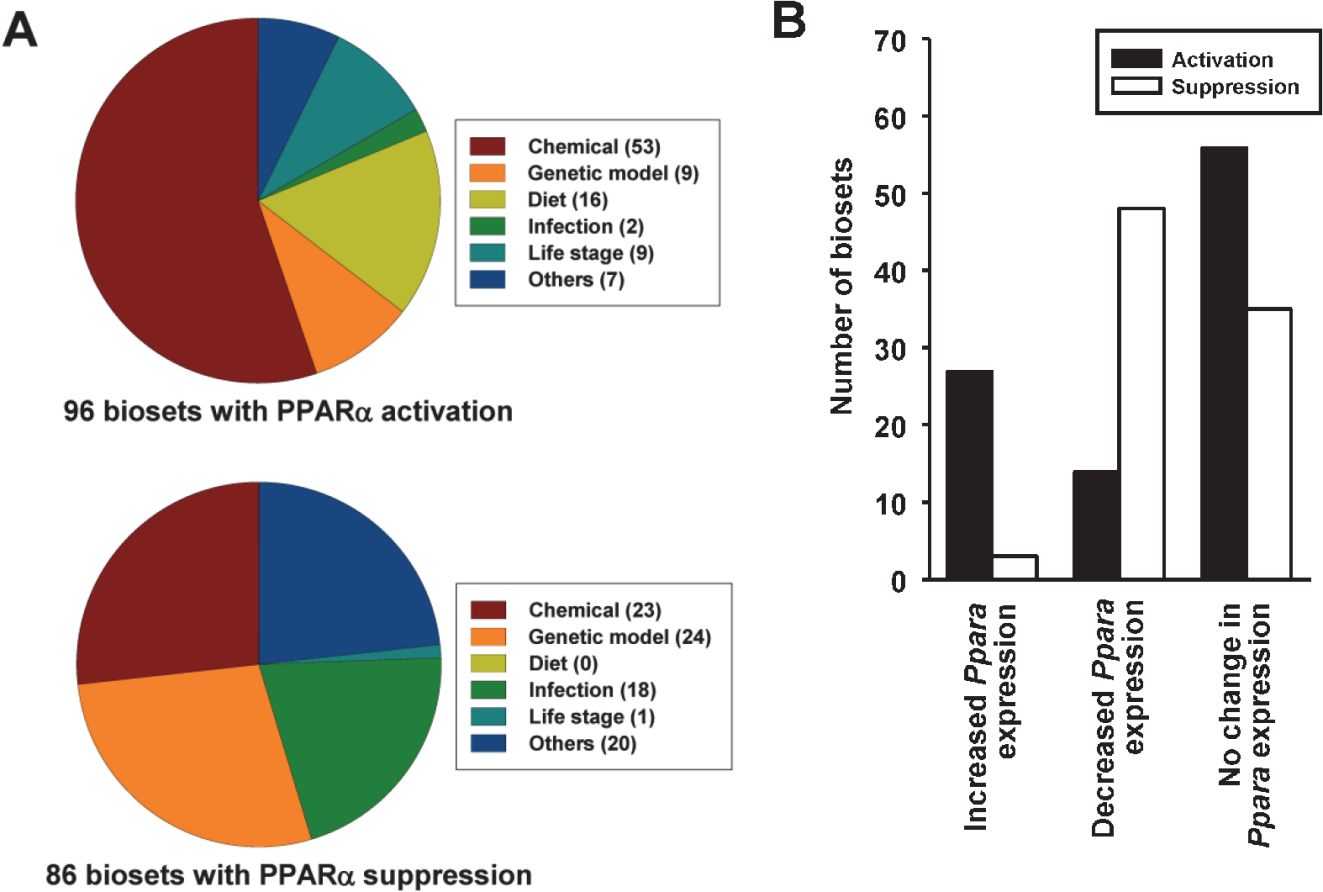
PPARα activation or suppression in a mouse liver compendium. A. Summary of PPARα activation or suppression. The PPARα signature was compared to ~1850 biosets using the Running Fisher’s test. The number of biosets with a p-value ≤ 10^-4^ for either activation or suppression in the indicated categories is shown. B. Relationships between *Ppara* expression changes and predictions of PPARα activity. The biosets were divided into those in which *Ppara* mRNA expression was increased, decreased or exhibited no change (∣fold-change∣ ≥ 1.2). Predictions of the number of biosets for PPARα activation or suppression are shown for the three groups.

Using the Running Fisher’s algorithm, PPARα was significantly altered in 182 biosets including 96 in which PPARα was activated and 86 in which PPARα was suppressed (p-value ≤ 10^-4^). The distribution of the biosets indicates that out of all of the factors examined, chemicals, genetic models, diets and infections have the largest numbers of effects on PPARα (**Fig. 5A**). The effects of chemicals, infections and diets on PPARα are discussed below. Because of space limitations the effects of specific gene mutations are discussed in another publication (Vasani et al., in preparation).

A number of mechanisms have been hypothesized that lead to activation or suppression of PPARα and regulated genes. Exposure to some PPC increase the expression of the *Ppara* gene itself and thus may augment regulation of target genes [4]. The relationships between changes in expression of *Ppara* and the signature predictions was determined (**Fig. 5B**). *Ppara* expression was derived from the statistically filtered microarray experiments of the same biosets evaluated for PPARα activity. The biosets were divided into those in which *Ppara* expression was increased, decreased or exhibited no change. For those biosets in which *Ppara* expression increased (fold-change ≥ 1.2), there were 127 biosets which exhibited no significant PPARα activation or suppression (p-value > 10^-4^) (not shown), 27 biosets in which PPARα was activated and only 3 biosets in which PPARα was suppressed. For those biosets in which *Ppara* expression was decreased, there were 219 biosets which exhibited no significant PPARα activation or suppression (p-value > 10^-4^) (data not shown), 14 biosets in which PPARα was activated and 48 biosets in which PPARα was suppressed. For those biosets in which there was no change in *Ppara* expression, there were 56 and 35 biosets that exhibited activation or suppression of PPARα, respectively. In summary, even though for a subset of biosets there was some concordance between *Ppara* expression and activation/suppression of PPARα, the expression of the *Ppara* gene does not appear to be tightly linked to PPARα activation.

### Chemical activation or suppression of PPARα

**Fig 6:**
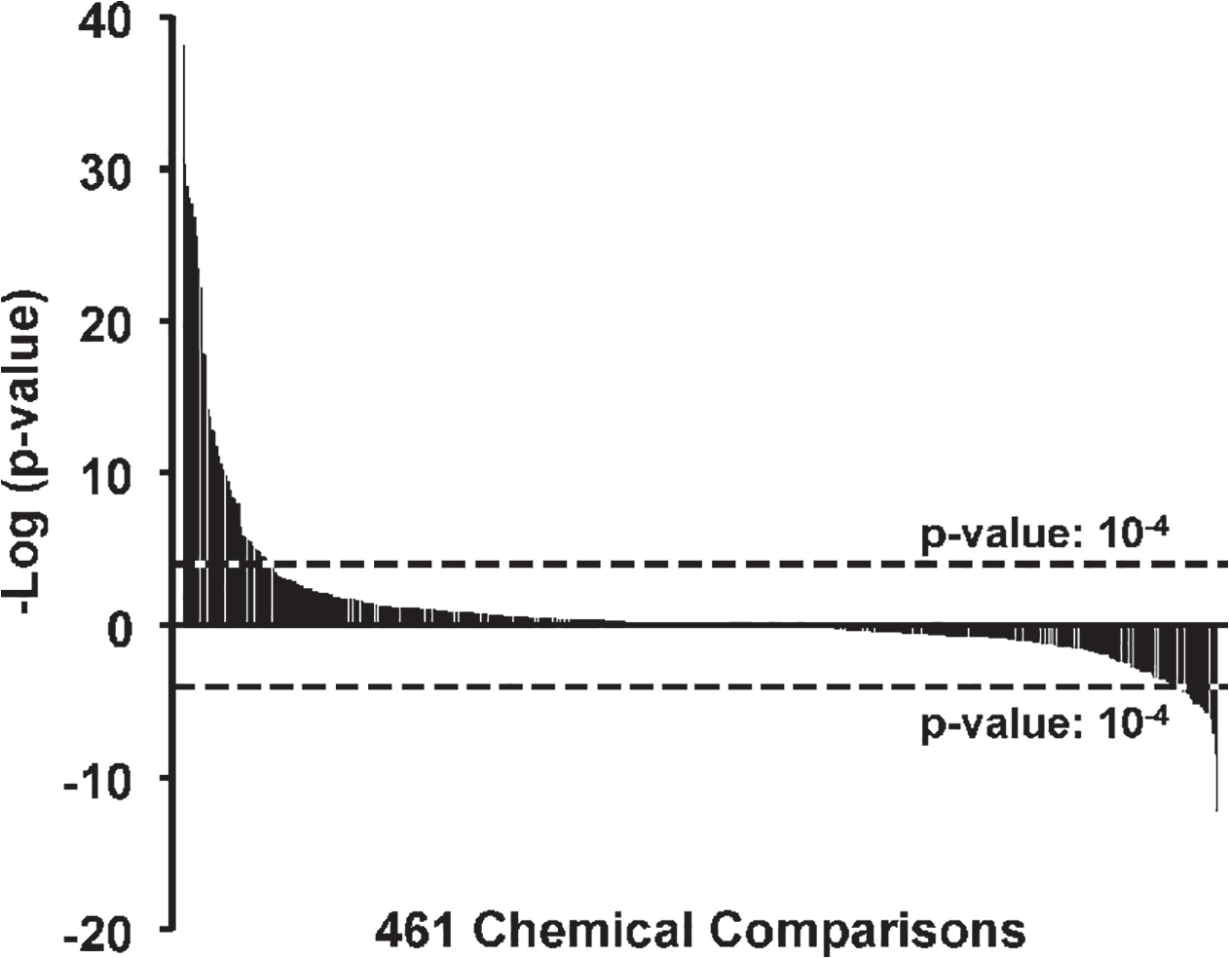
Chemical activation or suppression of PPARα. Distribution of chemical effects on PPARα activation or suppression. The Running Fisher’s test –log(p-values) for the 461 chemical comparisons are shown. The cutoff values for significance are shown.

The distribution of –log(p-values) of PPARα activation or suppression across 461 chemical comparisons representing ~150 chemicals is shown in Fig. 6; 53 of the chemical treatments significantly activated PPARα and 23 significantly suppressed PPARα. The entire list of chemical treatments that alter PPARα can be found in **S2 File**.

**Fig 7:**
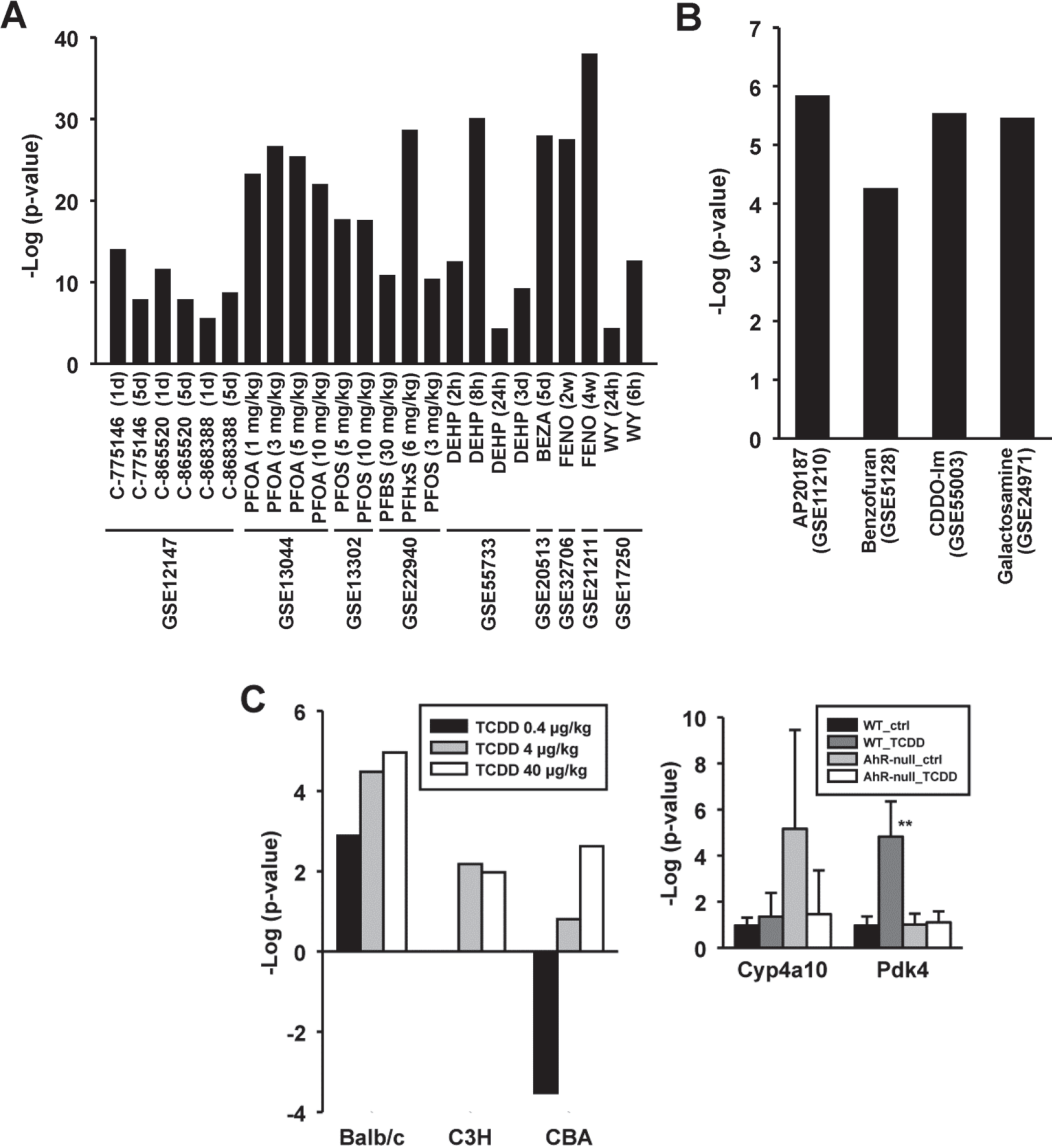
Activation of PPARα by diverse chemicals. A. Known activators of PPARα. B. Activation of PPARα by novel chemicals. Novel activators of PPARα identified in the screen are shown. C. Activation of PPARα by TCDD. (Left) Activation of PPARα by TCDD in Balb/c but not C3H or CBA mice at 4 or 40 μg/kg TCDD (from [47]). (Right) Expression behavior of PPARα-regulated genes in wild-type and AhR-null mice after exposure to TCDD. Significantly different from corresponding control: * p < 0.05, **p < 0.01. Significantly different between controls in wild-type and nullizygous mice: ^#^ p < 0.05, ^##^ p < 0.01.

In addition to the chemicals analyzed in the wild-type and PPARα-null mouse comparisons discussed above, a number of known activators of PPARα were found to activate PPARα in wild-type mice. These biosets included those from mice treated with piperidine-derived experimental hypolipidemic compounds, perfluorinated compounds including PFOA, PFOS, PFHxS, perfluorobutane sulfonic acid (PFBS), and DEHP, bezafibrate, fenofibrate and WY (**Fig. 7A and S2 File**).

Additional chemicals not commonly recognized as PPARα activators were also identified including AP20187, benzofuran, CDDO-Im, galactosamine, and TCDD (**Fig. 7B and C**).

**Fig 8:**
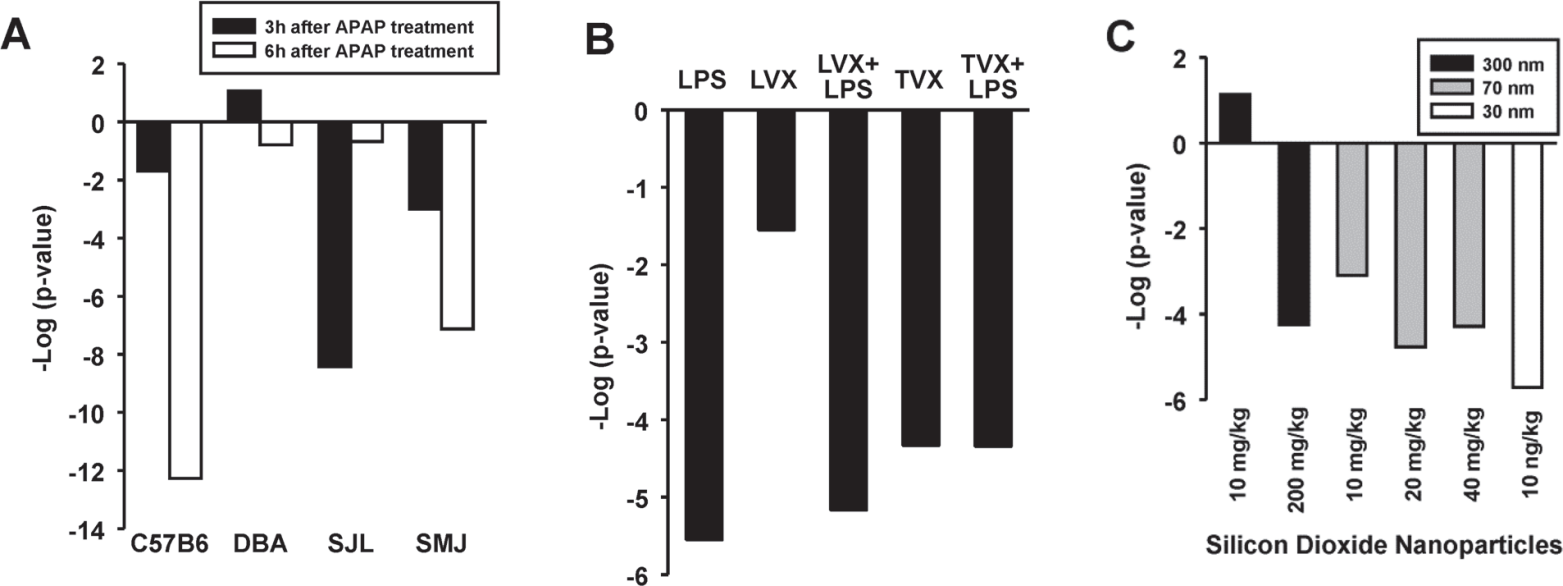
Suppression of PPARα by chemical exposure. A. Suppression of PPARα by acetaminophen. Effects of acetaminophen treatment were examined at either 3 or 6 hrs of exposure in four strains of mice [49]. B. Suppression of PPARα by LPS and trovafloxacin. Suppression of PPARα by LPS, LPS + lovafloxacin (LVX), trovafloxacin (TVX), and TVX + LPS but not by LVX only [51]. C. Suppression of PPARα by silicon dioxide nanoparticles. In general, suppression of PPARα was more significant with smaller particle size at equivalent dose levels [54].

- AP20187 is a compound used to stimulate the phosphorylation of the alpha subunit of translation initiating factor 2 (eIF2a). Dephosphorylation of eIF2a enhanced glucose tolerance and attenuated high fat diet induced hepatosteatosis [42].
- Benzofuran was a PPARα activator in one 13 week exposure study [43]. Derivatives of benzofuran such as benzbromarone activate PPARα-dependent markers in some test systems [44], but this is the first report of a link between benzofuran exposure and PPARα activation.
- The activation of PPARα by CDDO-Im occurred in Nrf2-null but not wild-type mice from Yates et al. (2009) [45].
- Mice which received galactosamine for 48 but not 24 hrs (GSE24971) exhibited significant PPARα activation. In a previous study, galactosamine exposure has been associated with suppression of PPARα expression and activity in a co-exposure model with LPS in KK-Ay obese mice [46].
- TCDD was found to increase PPARα activation in the Wu et al. study (2008) [47] in BALB/c but not C3H or CBA strains administered 4 or 40 μg/kg for 24 hrs (**Fig. 7C, left**). The marginal statistically significant p-values observed here are consistent with the modest increases in *Pdk4* expression in wild-type but not AhR-null mice after exposure to TCDD observed by RT-PCR (**Fig. 7C, right**). The changes in *Pdk4* were in contrast to the large increases in a marker of AhR activation (*Cyp1a1*) (data not shown). A number of other biosets from TCDD- or B[a]P-treated mice approached significance (data not shown). Recent reports have shown that AhR activators TCDD and B[a]P when given to mice result in disturbances of lipid homeostasis that led to steatosis ([48] and references therein). Based on these studies, we hypothesize that the other compounds identified here might activate PPARα secondarily to perturbation of lipid metabolism.
- Chemicals were identified that suppressed basal PPARα activity in the absence of known prior stimulation of PPARα activation.
- Transcriptional responses to acetaminophen (APAP)-induced liver injury were examined in 4 strains of mice for 3 or 6 hrs [49]. APAP suppressed PPARα in a strain- and time-dependent manner (**Fig. 8A**). The pattern of PPARα suppression somewhat paralleled the strain susceptibility to APAP-induced damage (C57 > SMJ >DBA > SJL) with strains most susceptible having the most significant PPARα suppression at 6 hrs. PPARα-regulated pathways have been shown to be targets of APAP treatment [50].

- The antibiotic trovafloxacin (TVX) has caused severe idiosyncratic hepatotoxicity in humans, whereas the structurally similar antibiotic levofloxacin (LVX) has not. Mice co-treated with TVX and lipopolysaccharide (LPS), but not with LVX and LPS, develop severe hepatocellular necrosis [51]. In a reanalysis of this study, it is shown here that LPS suppressed PPARα (**Fig. 8B**), consistent with a number of other reports of suppression by LPS [52, 53] (discussed below). Interestingly, in the absence of LPS, TVX but not LVX also caused suppression of PPARα, leading to the hypothesis that suppression of PPARα is linked to the mechanism of liver damage.
- Mice exposed intraperitoneally for 6 hrs to particles consisting of 3 different sizes of silicon dioxide caused suppression of PPARα (**Fig. 8C**). The pattern of suppression was somewhat consistent with the ability of the particle to cause liver damage, with smaller nanoparticles causing the greatest damage at equivalent doses [54] and the most significant suppression of PPARα.
- Additional chemicals that suppressed PPARα included epoxiconazole, myclobutanil, tert–butylhydroquinone, conconavalin A, malathion, bisphenol A, and *N*-ethyl-*N*-nitrosourea. Liver damage was reported after treatment with conconavalin A [55], consistent with an inflammatory state leading to PPARα suppression (discussed below). Bisphenol A was shown to suppress the mRNA level of the *Ppara* gene, increase expression of *Pparg*, and result in an accumulation of liver cholesteryl esters and triglycerides [56].

In conclusion, the PPARα signature was used to identify novel chemicals that activate or suppress PPARα. Further work will be required to determine if these effects on PPARα are through the parent compound and/or a metabolite and whether the effects are through direct binding or are indirect by altering the levels of endogenous metabolites that activate PPARα.

### Effects of infections on PPARα activation

**Fig 9:**
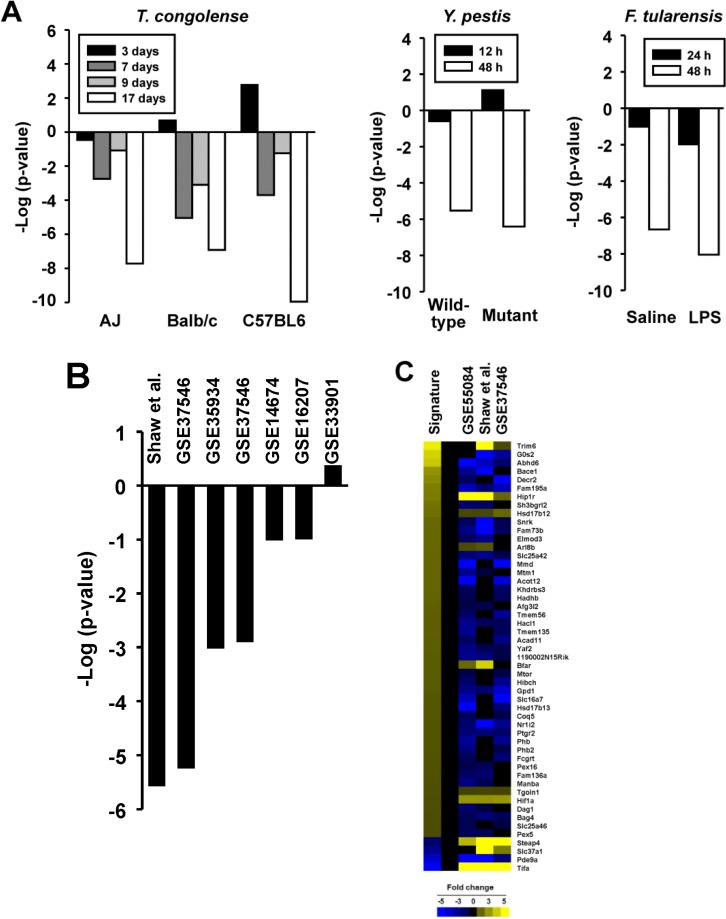
Suppression of PPARα by infections. A. Effect of infections on PPARα. (Left) Effect of *T*. *congolense* infection on PPARα. Different strains of mice were infected with *T*. *congolense* and examined for liver gene expression at 3, 7, 9, and 17d after infection [63]. (Middle) Effect of *Y*. *pestis* infection on PPARα. Mice were infected with 5 times the lethal dose 50 of wild-type or mutant form of *Y*. *pestis* and profiled either 12 or 48 hrs later [64]. (Right) Effect of *F*. *tularensis* infection on PPARα. Mice were infected with *F*. *tularensis* after LPS or saline pretreatment and profiled 24 or 48hrs later [57]. B. Suppression of PPARα by LPS. Seven biosets which examined effects of LPS on the liver transcriptome are shown. One bioset was not from GEO (Shaw et al., 2009; [51]). C. Heatmap showing the expression of genes after LPS exposure. Those biosets which exhibited significant suppression of PPARα are shown. The expression of genes that exhibited consistent expression (2 or 3 out of 3) are shown.

The 91 biosets from the livers of mice exposed to infectious agents were examined for effects on PPARα. A number of biosets derived from mice following infection showed suppression of PPARα. The species whose infection suppressed PPARα included *Trypanosoma congolense*, *Yersinia pestis*, and *Francisella tularensis* (**Fig. 9A**) as well as *Coxiella burnetii*, *Burkholderia pseudomallei*, *Ehrlichia chaffeenis* and *Streptococcus pneumonia* (data not shown). Suppression of PPARα was usually more severe with longer infection times. As *Francisella tularensis* and *Yersinia pestis* are gram negative bacteria, the effects of exposure to LPS (derived from gram negative bacteria) were evaluated. In addition to the bioset derived from female mice with significant suppression of PPARα (**Fig. 3D**), six of the 7 biosets in which transcriptional effects of bacterial LPS in the liver were examined in other studies showed suppression of PPARα (**Fig. 9B**), with two biosets exhibiting significant suppression including one discussed above (**Fig. 8B**) while 2 others approached significance. For the three biosets which exhibited significant suppression of PPARα after LPS exposure, 43 of the PPARα signature genes exhibited consistent behavior for suppression, i.e., decreased expression of genes positively regulated by PPARα and increased expression of genes negatively regulated by PPARα (**Fig. 9C**). In particular, there were strong increases in expression of three genes that were negatively regulated by PPARα (*Steap4*, *Slc37a1*, *Tifa*). A small set of genes (*Arl8b*, *Bfar*, *Hif1a*, *Hip1r*, *Hsd17b12*, *Tgoln1*, *Trim6*) were regulated in a manner similar to that in the signature across the treatments indicating that not all of the signature genes respond predictably to LPS exposure. Although there is at least one study showing the effects of infection on PPARα activation [57], the diversity of species shown here that affect PPARα has not been previously recognized. Further work is needed to determine how these infectious agents and LPS might suppress the activity of PPARα to regulate a subset of the PPARα signature genes.

### Effects of diets on PPARα activation

The effects of diet on PPARα are discussed in **S1 File**.

## Summary

A signature-based approach was used to identify chemicals, infections, and diets that have an impact on PPARα activity. To create and test the PPARα signature, we capitalized on microarray comparisons from the livers of wild-type and PPARα-null mice after exposure to either chemicals or synthetic triglycerides. Genes that were consistently activated or repressed in a PPARα-dependent manner were identified using a stringent set of criteria including 9 statistical tests and a number of filters ultimately resulting in a final list of 147 probe sets representing 131 genes. Most of these genes have been shown to be responsive to PPARα activators based on published studies annotated in the Comparative Toxicogenomics Database (CTD). Many of the genes included those involved in lipid homeostasis, as expected.

To screen for factors that lead to alterations of PPARα, we compared the signature to a gene expression compendium of annotated biosets using the fold-change rank-based nonparametric Running Fisher’s algorithm [27], analogous to the Gene Set Enrichment Analysis (GSEA) method [25, 26]. The signature reliably predicted PPARα activation. Our test to assess predictive capability gave a balanced accuracy of 98% (**Fig. 3F**) which was superior to predictions using a number of well known machine learning classification algorithms (**S1 File**). The signature was able to clearly distinguish between chemical activators of PPARα and activators of other xenobiotic-activated transcription factors (**Fig. 3D**). The methods used here have the additional advantage that factors that suppress PPARα can be simultaneously identified. The signature will be a useful starting point for identification of a smaller subset of genes that can be used to reliably predict effects in high-throughput screens of environmentally-relevant chemicals or drugs. Although these genes are applicable for screening chemicals in mouse liver or mouse hepatocyte-derived cell lines, it may be possible to use the signature in other tissues that exhibit relatively high expression of PPARα including heart, kidney, and muscle.

**Fig 10:**
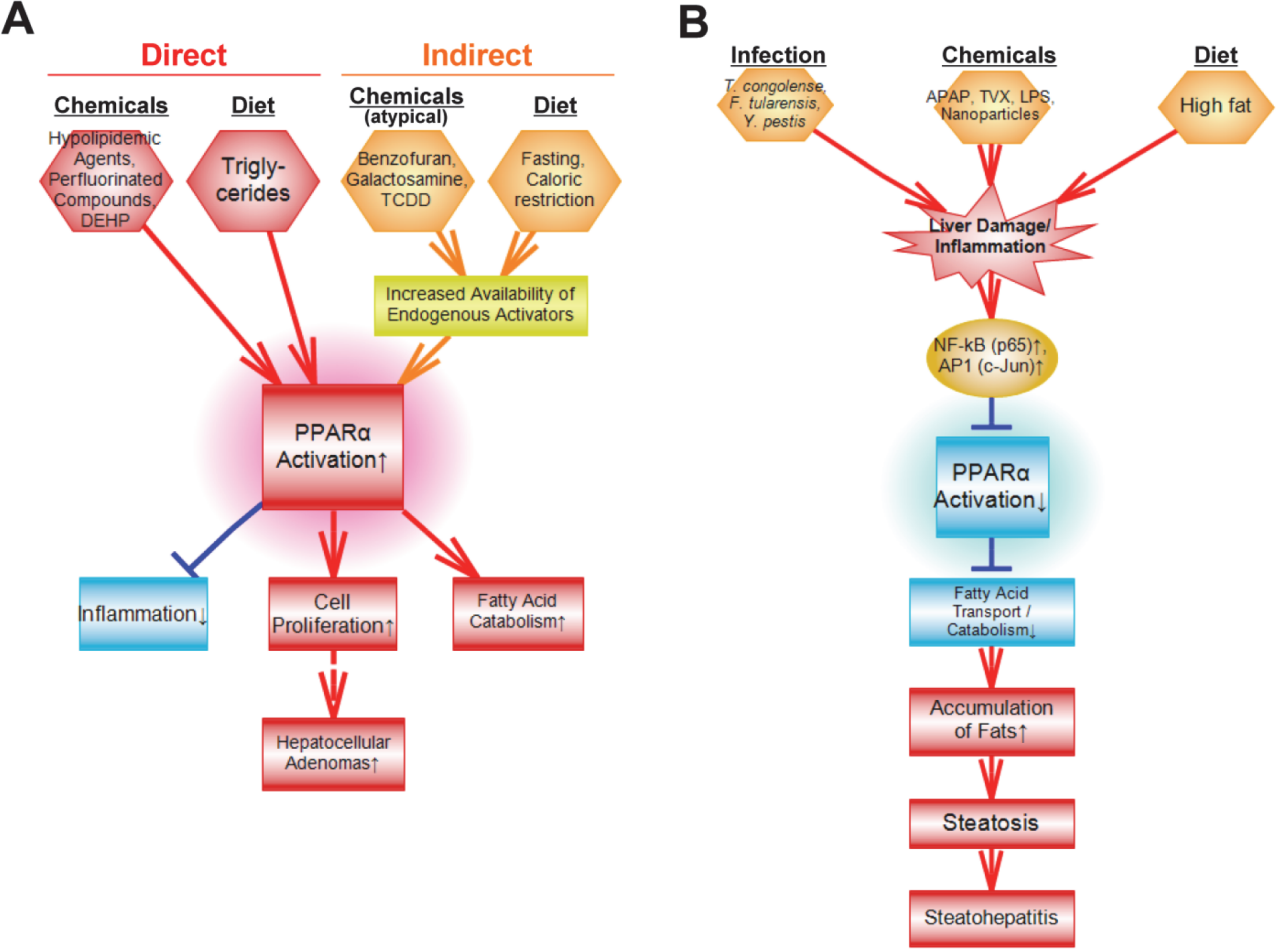
Summary of factors which affect PPARα. A. Summary of factors that activate PPARα. Although many chemicals and fatty acids (derived from hydrolysis of triglycerides) bind to and activate PPARα directly, some chemicals and diets likely activate PPARα indirectly by increasing the availability of endogenous PPARα activators. Downstream effects of PPARα activation include short-term responses of hepatocyte proliferation, activation of fatty acid transport and catabolism, and suppression of inflammatory responses. Under chronic exposure conditions, hepatocellular adenomas and carcinomas can develop. B. Summary of factors that suppress PPARα. A number of factors may suppress PPARα by increasing the activation of NF-kB and AP1 which physically interfere with the ability of PPARα to bind to DNA and regulate gene expression. Suppression of PPARα can lead to decreases in fatty acid catabolism and steatosis. Chronic suppression can lead to steatohepatitus.

Comparison of the PPARα signature to the liver gene expression compendium identified diverse factors that affect PPARα. A summary of the factors identified in our study which led to activation or suppression of PPARα is shown in **Fig. 10A, B**. Direct binding to PPARα by chemicals is the most widely recognized mechanism by which PPARα is activated. Typical activators of PPARα usually possess a bulky hydrophobic structure and an acidic group or a structural moiety that can be metabolized to an acidic group. The signature accurately predicted activation by compounds containing these structural features including hypolipidemic drugs, perfluorinated compounds, and synthetic triglycerides metabolized to fatty acid activators (**Figs. 3 and 7A**). A number of other compounds that do not possess these structural properties were found to activate PPARα. These atypical activators included benzofuran, galactosamine, the Nrf2 activator, CDDO-Im, AP20187 and the AhR activator, TCDD (**Fig. 7B, C**). We hypothesize that activation of PPARα by these chemicals is indirect, possibly through perturbation of fatty acid metabolism resulting in increases in endogenous activators of PPARα including fatty acids and eicosanoids. There is growing evidence that TCDD perturbs lipid metabolism and can increase levels of prostaglandin metabolites [58] that activate PPARα [59]. Other factors also activated PPARα including fasting and caloric restriction known to lead to mobilization of triglycerides in fat stores, transport to the liver, and catabolism by PPARα-regulated fatty oxidation genes (**S1 File**). Future efforts could focus on determining linkages between activation of PPARα and downstream events including lipid metabolism, cell fate and under long-term activation conditions, liver cancer.

Mechanisms by which chemicals or other treatments decrease PPARα activity are enigmatic. Decreases in activity could come about through decreases in the expression of the *Ppara* gene itself. However, decreases in *Ppara* gene expression do not consistently determine decreases in PPARα activation (**Fig. 5B**). The NF-kB subunit p65 (*Rela*) and the AP-1 subunit (*Jun*), both important mediators of inflammatory responses in multiple tissues, bind directly to the PPARα protein and inhibit activation at a PPRE [60, 61]. In addition, increasing amounts of MEKK which activates NF-kB caused decreases in the ability of PPARα to activate gene expression [62]. In vivo evidence linking inflammation with suppression of PPARα though is lacking. Our screen found a number of factors that decrease PPARα activity including chemicals (Fig. 8), infections (Fig. 9), and high fat diets (**S1 File**) that are all known to cause a cascade of effects including increases in liver injury, infiltration of inflammatory cells, secretion of cytokines, and induction of inflammatory mediators. The chemicals included acetaminophen, LPS, trovofloxacin, and nanoparticles. A subset of the high fat diets caused PPARα suppression presumably, because steatosis had progressed to steatohepatitis, although this hypothesis needs to be confirmed independently. A number of infectious agents associated with liver damage and inflammation also caused suppression of PPARα. Future studies could focus on the quantitative relationships between level of PPARα suppression and hypothesized phenotypic consequences of suppression of fatty acid catabolism and transport that can lead to steatosis and under prolonged exposure conditions, steatohepatitus.

A number of agencies that regulate chemicals have initiated programs to define the universe of AOPs affected by chemical exposure [1, 2]. These efforts are to place data from high throughput screens (e.g., EPA ToxCast screening program) into the context of AOP key events. The computational strategy described here, i.e., using the Running Fisher’s algorithm to find biosets with similarity to transcription factor signatures will be useful in future screens to characterize chemicals, diets, infections, etc. that impact key events in AOPs and in building cumulative risk models including those that predict liver cancer and steatosis.

## Supporting Information

S1 File Oshida et al.docx. Contains 1) treatment, dose, vehicle, and time of sacrifice of 12 treatment study in male and female mice; 2) classification accuracy of machine learning models used to predict PPARα activation; and 3) effects of diets on PPARα.
(DOCX)

S2 File Oshida et al.xlsx. Contains 1) list of probesets and genes in the PPARα signature; and 2) description of biosets that activate or suppress PPARα discussed in this study.
(XLSX)
