# Correction: Identification of Modulators of the Nuclear Receptor Peroxisome Proliferator-Activated Receptor α (PPARα) in a Mouse Liver Gene Expression Compendium

**DOI:** 10.1371/journal.pone.0124224

**Published:** 2015-04-07

**Authors:** The PLOS ONE Staff

There is an error in S2 File of the Supporting Information. The columns labelled “NextBio Bioset Name” and “Data Type” in S2 File are included erroneously. Please see the corrected S2 File here.

## Supporting Information

S2 File Supplementary dataset. Contains 1) list of probesets and genes in the PPARα signature; and 2) description of biosets that activate or suppress PPARα discussed in this study.
(XLSX)
