# Supplementary material for: Identification of Modulators of the Nuclear Receptor Peroxisome Proliferator-Activated Receptor α (PPARα) in a Mouse Liver Gene Expression Compendium
Source: PLoS One. 2015 Feb 17;10(2):e0112655. doi: 10.1371/journal.pone.0112655 (PMC4331523; doi:10.1371/journal.pone.0112655)
Supplement: S1 File — Contains 1) treatment, dose, vehicle, and time of sacrifice of 12 treatment study in male and female mice; 2) classification accuracy of machine learning models used to predict PPARα activation; and 3) effects of diets on PPARα. (DOCX) [file pone.0112655.s001.docx]

**Supplemental File 1.**

**Treatment, dose, vehicle, and time of sacrifice of 12 treatment study in male and female mice (Study 3).**

|  | | | | |
| --- | --- | --- | --- | --- |
|  |  |  | |  |
| Treatment | Dose^a^ | Vehicle | Amount | Time of Sacrifice |
| Aroclor 1260 | 200 mg/kg | Corn oil | 10 ml/kg | 48 h |
| β-Naphthoflavone | 50 mg/kg | Corn oil | 10 ml/kg | 24 h |
| Ciprofibrate | 250 mg/kg (gavage) | DMSO | 10 ml/kg | 8 h |
| Cobalt chloride | 60 mg/kg | 0.9% saline | 10 ml/kg | 48 h |
| TCDD | 10 μg/kg | DMSO | 100 μl/kg | 48 h |
| IL-6 | 25 μg/kg | PBS | 10 ml/kg | 6 h |
| Lipopolysaccharide | 1 mg/kg | PBS | 10 ml/kg | 6 h |
| PCB-153 | 80 mg/kg | Corn oil | 10 ml/kg | 48 h |
| Phenobarbital | 100 mg/kg/day (3 days) | 0.9% saline | 10 ml/kg | 72 h |
| Phenylhydrazine | 100 mg/kg | PBS | 10 ml/kg | 48 h |
| TNFα | 50 μg/kg | PBS | 10 ml/kg | 6 h |
| WY-14,643 | 250 mg/kg (gavage) | DMSO | 10 ml/kg | 8 h |
| ^a^Dose administered i.p. unless otherwise noted. | | | | |

**Classification accuracy of machine learning models used to predict PPARα activation.**

**Classification results using both wild-type and PPARα-null samples.**

|  | CCP | LDA | 1NN | 3NN | NC | SVM | BCCP |
| --- | --- | --- | --- | --- | --- | --- | --- |
| Sensitivity (%) | 100 | 100 | 98.5 | 98.5 | 100 | 98.5 | 100 |
| Specificity (%) | 69.2 | 67.4 | 68.3 | 75.9 | 73.7 | 67.9 | 72.4 |
| Balanced accuracy (%) | 76.2 | 74.8 | 75.2 | 81.0 | 79.7 | 74.8 | 79.7 |

**Classification results using only wild-type samples.**

|  | CCP | LDA | 1NN | 3NN | NC | SVM | BCCP |
| --- | --- | --- | --- | --- | --- | --- | --- |
| Sensitivity (%) | 97.0 | 92.4 | 98.5 | 100 | 98.5 | 98.5 | 98.4 |
| Specificity (%) | 68.3 | 66.1 | 60.5 | 61.4 | 77.4 | 69.9 | 74.3 |
| Balanced accuracy (%) | 73.2 | 70.6 | 67.0 | 68.1 | 81.0 | 74.8 | 78.8 |

CCP: Compound Covariate Predictor, LDA: Diagonal Linear Discriminant Analysis, 1NN: 1-Nearest Neighbor Classifications, 3NN: 3-Nearest Neighbor Classifications, NC: Nearest Centroid, SVM: Support Vector Machines with linear kernel, BCCP: Bayesian Compound Covariate Predictor.

**Effects of diets on PPARα activation.**

In addition to the synthetic triglycerides discussed in the main body of this study, there were a modest number of dietary manipulations that either activated or suppressed PPARα (11 or 9 out of 220 biosets, respectively). PPARα is activated by mobilized triglycerides during times of starvation [1-3]. Two of the 18 contrasts from mice that were calorically restricted and 4 of the 19 contrasts from mice that were fasted showed activation of PPARα (p-value ≤ 0.0001) (**Supplemental Figure 1A, B**). Many of the other contrasts approached significance and none of the contrasts showed significant suppression of PPARα. Differences in activation may be due to differences in level of food restriction and genetic background. The genes that were consistently altered in the four significant fasting biosets are shown in **Supplemental File 1C**. Most of these genes exhibited behavior similar to that in the signature. However, there were 4 genes (*Hsd17b12, Mtap, Pcyox1, Qrsl1*) that exhibited behavior opposite to the signature, indicating these genes may be regulated under fasting conditions by transcription factors other than PPARα.

Additional diets led to PPARα activation. A fish oil containing diet [4] and a high calorie diet [5] also activated PPARα. Three diets in one study [6] activated PPARα including cooked food from the Institute’s cafeteria (Café diet), McDonald's fast food (fast food diet) and a diet consisting of vegetables, fruit and yogurt identical to the diet fed to chimpanzees (Chimp diet), all compared to a mouse pellet diet on which the mice were raised (data not shown). The authors noted gene expression differences between the diets but did not determine the molecular basis for the differences.

There were 98 biosets that examined the effects of a high fat diet verses a “normal” diet. A number of these studies resulted in suppression of PPARα [7-9], whereas none of the high fat diets resulted in significant PPARα activation (**Supplemental Figure 1D**). The genes that were consistently altered by the high fat diets are shown in **Supplemental Figure 1E** for the 8 biosets which exhibited significant PPARα suppression. Most of the genes exhibited changes in expression that were opposite to that in the signature. Other genes were either not affected or exhibited expression similar to that in the signature (*1190002N15Rik, Anxa2, Dcp1a, Lpl, Pcyox1, Qrsl1*). The reason why a high fat diet can suppress PPARα activity is not known but might be related to the inflammatory state of steatohepatitis. Most, if not all of the high fat diets caused steatosis as expected (see individual studies listed in **Supplemental File 2**). There is some evidence from these studies that the liver progressed to steatohepatitis. Some liver damage was apparent in the Ishimura et al. study [7] as ALT levels were elevated in the high fat diet groups after 6 weeks. Shockley et al. [9] showed that high-fat feeding induced genes in the immune response, indicating inflammatory infiltration; however, no measures of liver damage were examined in this study. Thus, it remains to be determined whether there is definitive steatohepatitis in these studies that can be linked to suppression of PPARα. In conclusion, we found that fasting, caloric restriction, fish oil and certain diets activated PPARα while in some cases, high fat diets suppressed PPARα.


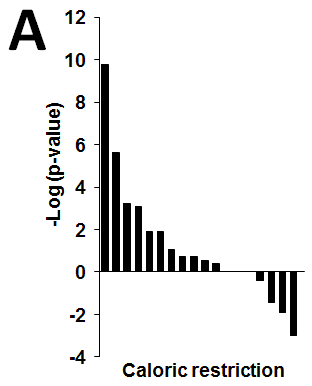

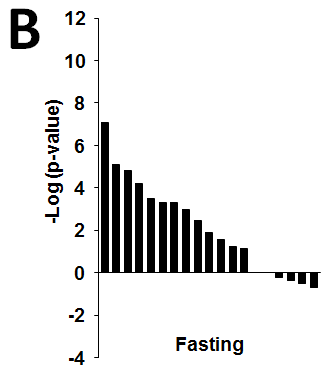

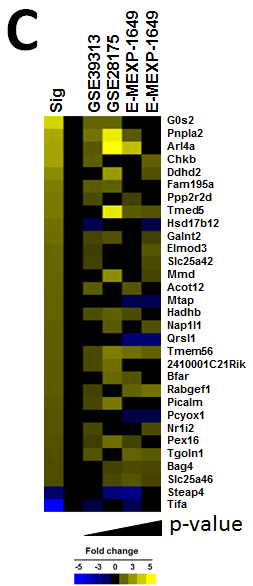

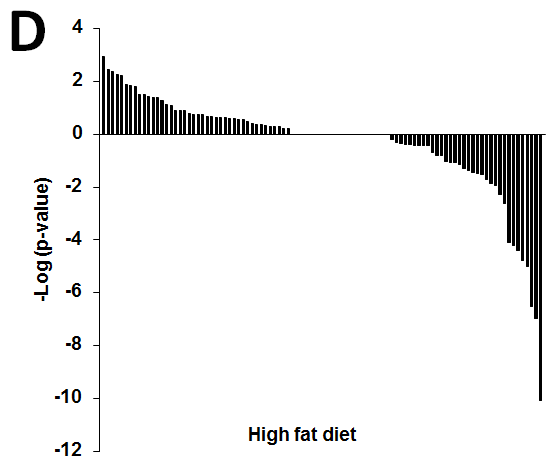


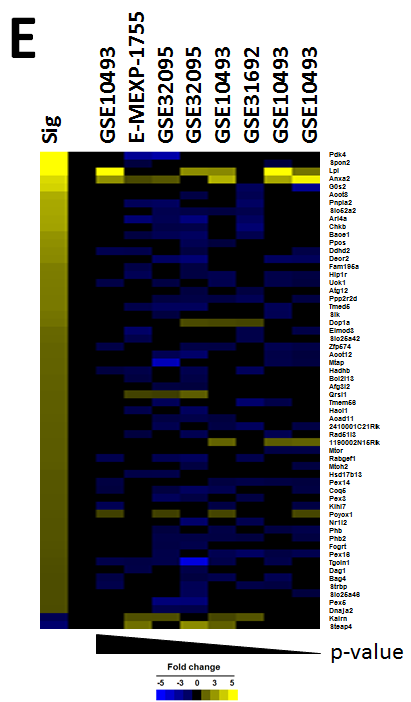


**Supplemental Figure 1. Effect of dietary manipulations on PPARα.**

A. Activation of PPARα by caloric restriction.

B. Activation of PPARα by fasting.

C. Heatmap showing the expression of genes after fasting in biosets with significant activation of PPARα. The expression of signature genes that exhibited consistent expression across the 4 biosets (2 or more out of 4) are shown.

D. Effects of high fat diets on PPARα.

E. Heatmap showing the expression of the PPARα signature genes after high fat diets in 8 biosets with significant suppression of PPARα. Only genes that exhibited consistent expression across the biosets (2 or more out of 8) are shown.

**References**

1. Hashimoto T, Cook WS, Qi C, Yeldandi AV, Reddy JK, et al. (2000) Defect in peroxisome proliferator-activated receptor alpha-inducible fatty acid oxidation determines the severity of hepatic steatosis in response to fasting. J Biol Chem 275: 28918-28928.

2. Kersten S, Seydoux J, Peters JM, Gonzalez FJ, Desvergne B, et al. (1999) Peroxisome proliferator-activated receptor alpha mediates the adaptive response to fasting. J Clin Invest 103: 1489-1498.

3. Leone TC, Weinheimer CJ, Kelly DP (1999) A critical role for the peroxisome proliferator-activated receptor alpha (PPARalpha) in the cellular fasting response: the PPARalpha-null mouse as a model of fatty acid oxidation disorders. Proc Natl Acad Sci U S A 96: 7473-7478.

4. Lu Y, Boekschoten MV, Wopereis S, Muller M, Kersten S (2011) Comparative transcriptomic and metabolomic analysis of fenofibrate and fish oil treatments in mice. Physiol Genomics 43: 1307-1318.

5. Baur JA, Pearson KJ, Price NL, Jamieson HA, Lerin C, et al. (2006) Resveratrol improves health and survival of mice on a high-calorie diet. Nature 444: 337-342.

6. Somel M, Creely H, Franz H, Mueller U, Lachmann M, et al. (2008) Human and chimpanzee gene expression differences replicated in mice fed different diets. PLoS One 3: e1504.

7. Ichimura A, Hirasawa A, Poulain-Godefroy O, Bonnefond A, Hara T, et al. (2012) Dysfunction of lipid sensor GPR120 leads to obesity in both mouse and human. Nature 483: 350-354.

8. Toye AA, Dumas ME, Blancher C, Rothwell AR, Fearnside JF, et al. (2007) Subtle metabolic and liver gene transcriptional changes underlie diet-induced fatty liver susceptibility in insulin-resistant mice. Diabetologia 50: 1867-1879.

9. Shockley KR, Witmer D, Burgess-Herbert SL, Paigen B, Churchill GA (2009) Effects of atherogenic diet on hepatic gene expression across mouse strains. Physiol Genomics 39: 172-182.
